# cognac: rapid generation of concatenated gene alignments for phylogenetic inference from large, bacterial whole genome sequencing datasets

**DOI:** 10.1186/s12859-021-03981-4

**Published:** 2021-02-15

**Authors:** Ryan D. Crawford, Evan S. Snitkin

**Affiliations:** 1 Department of Computational Medicine and Bioinformatics, University of Michigan, Ann Arbor, MI 48109 USA; 2 Department of Microbiology and Immunology, University of Michigan, Ann Arbor, MI 48109 USA

**Keywords:** Concatenated gene tree, Core genome, Multiple sequence alignment, Phylogenetics

## Abstract

**Background:**

The quantity of genomic data is expanding at an increasing rate. Tools for phylogenetic analysis which scale to the quantity of available data are required. To address this need, we present cognac, a user-friendly software package to rapidly generate concatenated gene alignments for phylogenetic analysis.

**Results:**

We illustrate that cognac is able to rapidly identify phylogenetic marker genes using a data driven approach and efficiently generate concatenated gene alignments for very large genomic datasets. To benchmark our tool, we generated core gene alignments for eight unique genera of bacteria, including a dataset of over 11,000 genomes from the genus *Escherichia* producing an alignment with 1353 genes, which was constructed in less than 17 h.

**Conclusions:**

We demonstrate that cognac presents an efficient method for generating concatenated gene alignments for phylogenetic analysis. We have released cognac as an R package (https://github.com/rdcrawford/cognac) with customizable parameters for adaptation to diverse applications.

## Background

Phylogenetic analysis is becoming an increasingly integral aspect of biological research with applications in population genetics, molecular biology, structural biology, and epidemiology [1]. Generating a quality multiple sequence alignment (MSA) is fundamental to robust phylogenetic inference. MSA is a foundational tool in many disciplines of biology, which aims to capture the relationships between residues of related biological sequences, and therefore facilitate insights into the evolutionary or structural relationships between the sequences in the alignment.

The first analysis incorporating genetic sequences to understand the evolutionary history of an organism was a sample of 11 *Drosophila melanogaster* Adh alleles in 1983 [2]. Since then, there has been a growing interest in using gene sequences to estimate the evolutionary relationships between organisms. However, it was quickly observed that individual gene trees are often inaccurate estimations of the species tree [3]. These incongruencies can arise from errors while building the tree, or from biological processes such as incomplete lineage sorting, hidden parology, and horizontal gene transfer [4].

One approach for mitigating the incongruence between gene and species trees is the analysis of multiple genes at multiple loci concatenated into a supergene to generate more precise phylogenies [5–9]. This approach better leverages the large quantity of available data using multiple genes to substantially increase the number of variant sites and minimize the stochastic errors that may be associated with the limited information contained in a single gene [10]. This approach to infer the species tree has also been shown to be accurate under a range of simulated conditions, in spite of the biological processes which might pose a challenge to accurate phylogenetic inference [11, 12].

Prior selection of a gene or set of genes for a given species is a commonly used strategy for selecting phylogenetic marker genes. For bacteria, the most commonly used marker gene for phylogenetic analysis is the 16S rRNA gene [13]. This gene is ubiquitous in bacteria and archaea with highly conserved and variable regions which makes it a useful marker for estimating the evolutionary relationships between prokaryotes; however, this gene evolves slowly, often resulting in few variant positions within a species. Curated methods for selecting marker genes, such as multi-locus sequence typing, expand the number of marker genes for a given species, and have led to improved resolution within a species [14]. However, this approach remains limited in that only a small number of curated genes are selected for a specific species, limiting its application to understudied organisms. Recently this concept has been expanded to include 400 marker genes commonly present in bacteria and archaea concatenated into a supergene for phylogenetic analysis of prokaryotes [10]. While these tools have many useful applications, relying on a limited number of predefined genes may limit the number of phylogenetically informative markers contained in a given dataset, which is important in situations where maximizing variation to distinguish closely related isolates is required.

In this work, we present cognac (core gene alignment concatenation), a novel data-driven method and rapid algorithm for identifying phylogenetic marker genes from whole genome sequences and generating concatenated gene alignments, which scales to extremely large datasets of greater than 11,000 bacterial genomes. Our approach is robust when handling data sets with extremely diverse genomes and is capable of creating an alignment with large numbers of variants for phylogenetic inference.

## Implementation

**Fig. 1:**
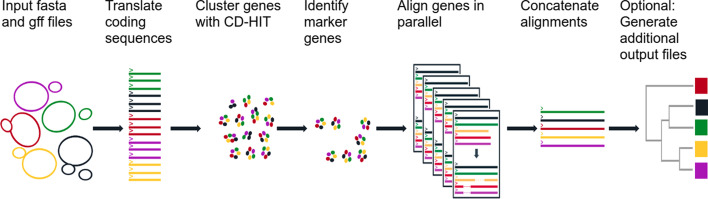
Overview of the cognac algorithm. Whole genome sequences and gene annotations are input, and the coding sequences are extracted and translated to return the amino acid sequences. The amino acid sequences are clustered to identify orthologous genes and the single copy, core genes are extracted from the dataset. For each core gene, unique alleles are identified and aligned and the alignment is parsed to represent the aligned sequence for the full dataset. Alignments are then concatenated, and are ready for downstream analysis

The inputs to cognac are fasta files and genome annotations in gff format, which can be obtained via commonly used programs such as, RAST, Prokka, or Prodigal (Fig. 1) [17–19]. First, the sequences corresponding to the coding genes are extracted using the coordinates provided in the gff file, and the nucleotide sequence for each gene is translated. To identify phylogenetic marker genes, CD-HIT is then used to cluster the amino acid sequences into clusters of orthologous genes (COGs) by their sequence similarity and length [20]. By default, COGs are defined at a minimum of 70% amino acid identity, and that the alignment coverage for the longer sequence is 80% at minimum.

The CD-HIT output file is then parsed and marker genes within the dataset are selected for inclusion in the alignment [20]. By default cognac identifies core genes to a given set of genomes; however, the selection criteria are customizable to allow for flexibility when creating alignments for various applications. The default selection criteria for selecting marker genes are: 1) present in 99% of genomes, 2) present in a single copy in 99.5% of genomes, and 3) ensuring that there are at least one variant position in the gene sequence. Allowing some degree of missingness allows for assembly errors which may arise in large datasets. We also allow the user to input a minimum number of genes to be included, and a minimum fraction of genes which are allowed to be missing, as genomes that don’t share a sufficient number of phylogenetic markers may be problematic for some types of phylogenetic analysis and/or be indicative of problematic samples.

Once the marker genes are identified, the individual gene alignments of the amino acid sequences for each gene are generated with MAFFT [21]. Prior to alignment, redundant sequences of each gene are identified, and only the unique alleles are input to MAFFT. In particular, for each gene identified by CD-HIT, we first look for exact string matches within each gene cluster and select the representative unique alleles. The unique alleles are input to MAFFT and the amino acid alignment is generated. The output gene alignment is then parsed, replicating the aligned sequence corresponding to each duplicated allele, generating the alignment for the entire set of alleles. Because MSA is computationally intensive, minimizing the number of sequences to align helps to reduce the associated computational overhead, leading to significantly reduced memory consumption and run-time.

Finally, the individual genes are concatenated into a single alignment to be used in downstream analysis. The alignment can then be input to commonly used programs for generating phylogenetic trees, such as RaXML or FastTree to create a maximiumium likelihood (ML) tree or approximate ML tree respectively. We have included the ability to directly generate a neighbor joining tree within the R package, to allow users to easily create a tree. cognac is well suited to generating alignments for extremely large datasets, and in these instances the computational workload for ML based methods may be prohibitive, and therefore creating a neighbor joining tree may present a good option. Neighbor joining trees are a distance based method which require a much less computational overhead relative to ML based methods. While ML methods are likely to produce better results, the increased speed may be desirable for situations where a high degree of precision is not required.

Additionally, several optional outputs may be generated. We provide the functionality to generate a nucleotide alignment by mapping the corresponding codons to the amino acid alignment. We use gap placement in the amino acid alignment to position the corresponding codons from the nucleotide sequence of each gene, generating a codon aware nucleotide alignment. This has the added benefit of increasing the number of variant positions in the alignment, which are a product of synonymous substitutions. This is potentially useful for applications where maximizing variation is key. We also provide functionality for parsing the alignments including: eliminating gap positions, removing non-variant positions, partitioning the alignment into the individual gene alignments, removing low quality alignment positions, and creating distance matrices.

Cognac was developed for R version 4.0.2. C +  + code was integrated via the Rcpp package (version 1.0.3) and was written using the C +  + 11 standard [22]. Multithreading is enabled in the C +  + code via RcppParallel, which provides wrapper classes for R objects used by Intel Threading Building Blocks parallel computing library [23]. Multithreading for R functions was enabled via the future.apply package (version 1.3.0) [24]. Functions for analysis of phylogenetic trees were enabled via the APE R package (version 5.3). [25].

## Results

To demonstrate the utility of our tool, we created genus-level core gene alignments for 27,529 genomes from eight clinically relevant species of bacteria (Table 1, Additional file 1: Table S1). The number of genomes included from each genera had a wide range from 24 for *Pluralibacter* to 11,639 for *Escherichia*. Cognac was run requiring that at least 1000 genes which qualify as core genes included in the alignment and genomes missing greater than 1% of core genes were removed. This was a large data set with the potential for inaccurate species assignment or assemblies to be of poor quality, ensuring that these genomes do not limit the number of core genes included. Additionally, for our test runs we included the optional steps to generate the nucleotide alignment, create a pairwise single nucleotide variant distance matrix from the nucleotide alignment, and generate a neighbor joining tree.

**Table 1:** Description of dataset and run statistics for the analysis in this study

	Number of genomes	Total number of coding sequences	Number of core genes	Alignment length (amino acid residues)	Run time (min)	Memory usage (GB)
*Citrobacter*	262	1,356,975	1864	590,749	14.78	3.4
*Enterobacter*	1947	9,575,752	1671	551,522	105.81	37.39
*Escherichia*	11,639	6,104,2774	1353	387,857	693.38	223.81
*Klebsiella*	9879	55,944,623	1957	631,196	980.95	184.86
*Pluralibacter*	24	131,798	1919	611,547	2.88	1.21
*Proteus*	207	806,518	1081	305,078	4.94	2.41
*Pseudomonas*	3051	19,509,251	1065	313,694	95.42	47.83
*Serratia*	520	2,673,835	1109	327,628	14.1	6.84

**Fig. 2:**
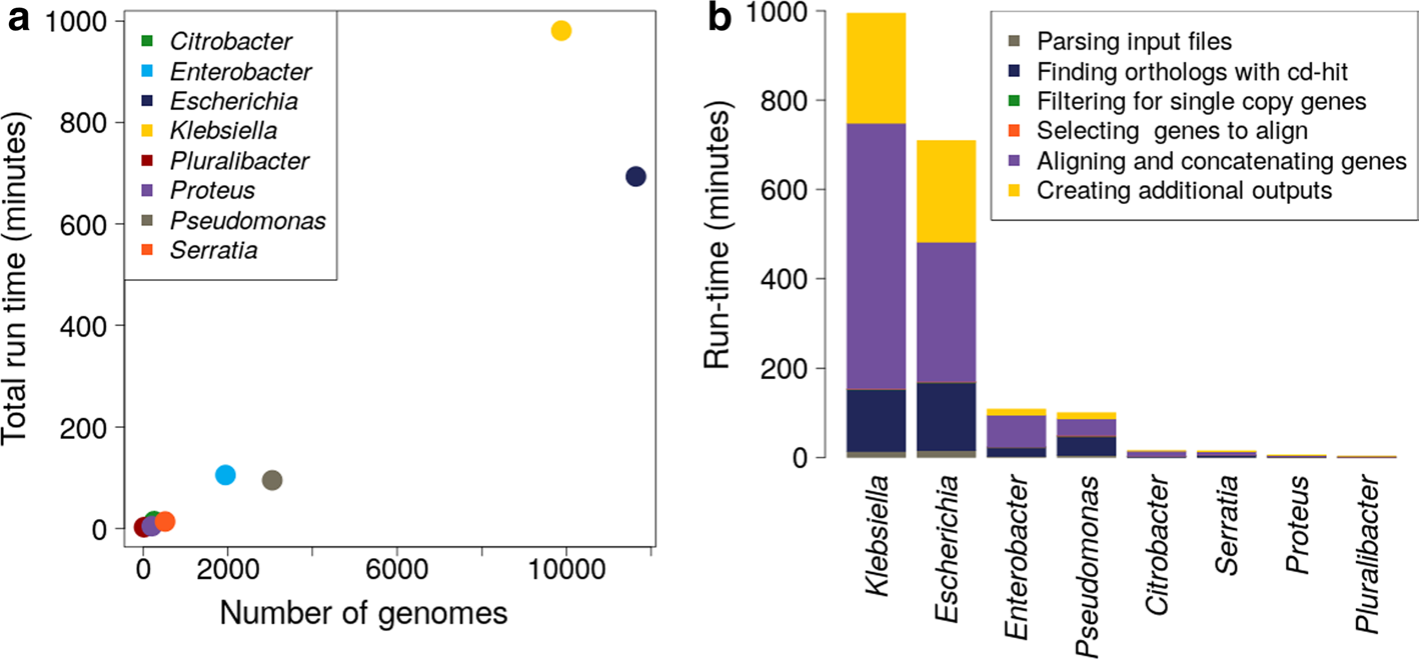
cognac is able to maintain reasonable run time even for very large datasets, generating the amino acid alignment, mapping back to nucleotide sequence, creating a distance matrix, and neighbor joining tree. a For each genus the run time plotted against the number of genomes included in the analysis. b The composition of the run time by step

All runs finished in less than a day, and ranged from three minutes to 16 h and 21 min (Table 1). Run-time grew linearly as the number of genomes increased (Fig. 2a). For all runs, with the exception of *Pseudomonas*, generating the MAFFT alignments was the largest portion of the total run-time (Fig. 2b). The CD-HIT step was the highest fraction of runtime for *Pseudomonas* due to the larger genome size and the large degree of pan-genome diversity observed for this genus (Table 1).

**Fig. 3:**
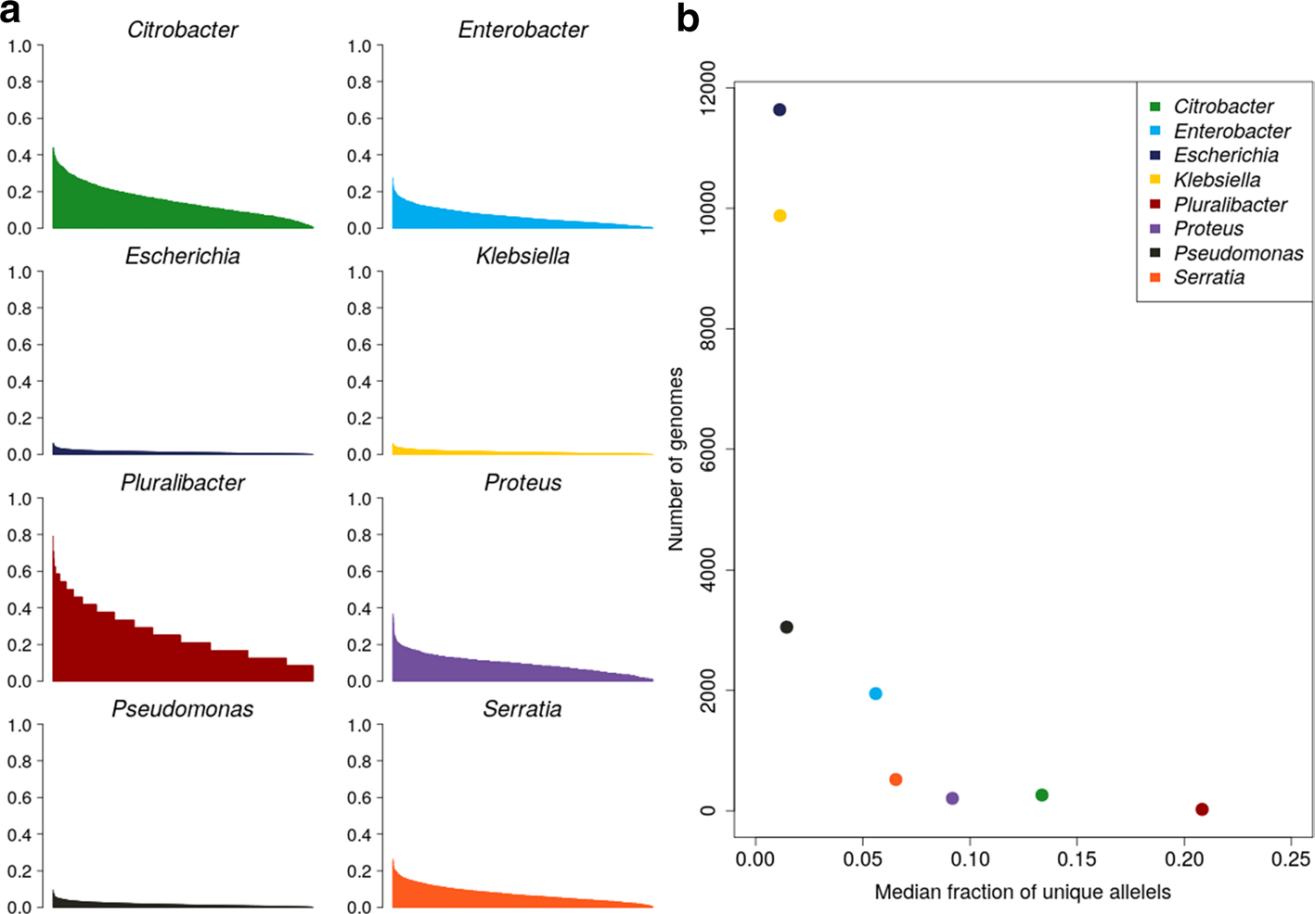
The fraction of unique alleles per gene is inversely proportional to the number of genomes in the dataset. **a** The distribution of unique alleles per core gene included in the alignment as a fraction of the number of genomes. **b** The relationship between the number of genomes and the median fraction of unique alleles for each gene

To assess the magnitude of the reduction in the quantity of sequences that were aligned by selecting only the unique alleles of each gene, which is related to increased computational efficiency, we calculated the number of unique alleles per core gene as a fraction of the number of genomes (Table 1, Fig. 3a). We observed a strong inverse relationship between the number of genomes included and the number of unique alleles identified within the dataset (Fig. 3b). As a fraction of the number of genomes, *Klebsiella* had the lowest range of unique alleles with 0.02% (n = 2) to 6.07% (n = 600), with a median of 1.13% (n = 112). *Pluralibacter* had the fewest genomes and had the highest proportion of unique alleles, with a maximum value of 79.9% unique alleles (n = 19). This is a substantial decrease in the quantity of sequences that need to be aligned, enabling cognac to scale to very large datasets. Because organisms are related genealogically, sequences in the genome are not independent, sharing a common ancestor. Therefore adding additional genomes does not necessarily expand the number of unique alleles for any genes, and all of the sequences may be represented by a substantially reduced subset of the number of samples.

**Fig. 4:**
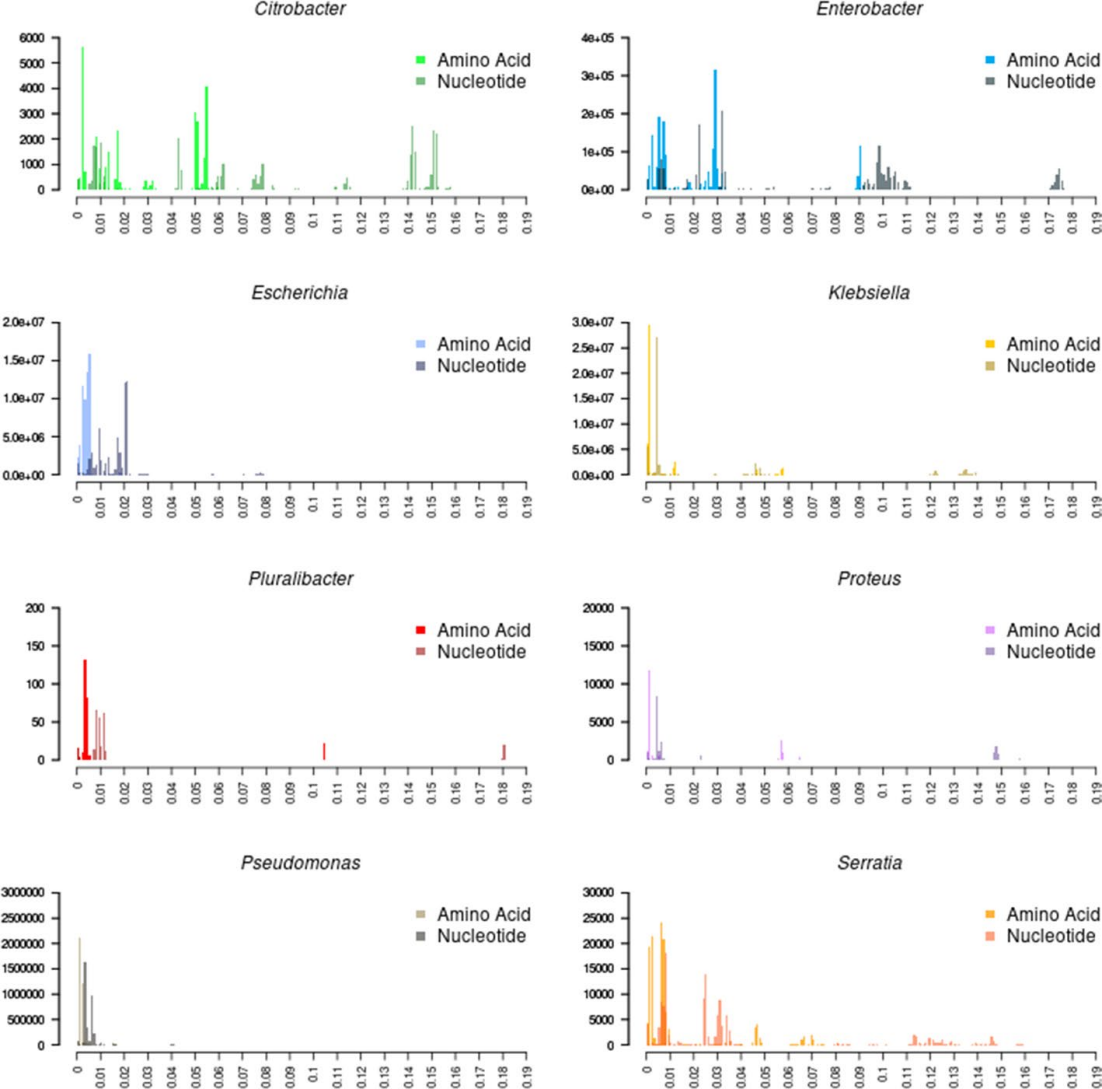
Mapping back to the nucleotide sequence of core gene alignments expands the number of variants for phylogenetic analysis. Pairwise distance matrices were constructed with the raw number of substitutions of the amino acid and nucleotide alignments. Histograms show the distribution of substitutions per position in the alignment. Lighter colors represent the amino acid distances and darker colors represent the nucleotide distances

We then wanted to analyze the effect of converting the amino acid alignments to nucleotide alignments with respect to amplifying the sequence diversity. The raw number of pairwise substitutions was calculated between all genomes from both the amino acid alignment and nucleotide normalized to the alignment length (Fig. 4). This greatly expanded the quantity of genetic variation contained in the alignment, although to different degrees for different datasets. This may reflect non-biological processes. For example, different data sets may have more diversity due to non-random sampling of the diversity within each genus. Additionally, the magnitude of the phylogenetic distances between isolates may not be uniform within different taxonomic assignments. Although biological factors may also play a role in the observed genetic distances. For example, the lowest amount of diversity was observed in *Pseudomonas*. The published mutation rate for *E. coli* is 2.5 times higher than that of *P. aeruginosa,* suggesting that the differences in diversity may be a function of the mutation rate in these organisms [26].

## Discussion

We present a method to rapidly identify over 1000 marker genes and generate concatenated gene alignments that is capable of handling diverse bacterial genomes. Recently, we used this method to generate a core genome alignment and maximum likelihood tree for 52 genomes in the family *Bacteroidetes*, illustrating the utility of this tool to create gene trees over large phylogenetic distances [27]. Importantly, phylogenetically informative marker genes are selected using a data driven approach, without any knowledge of the input genomes a priori, which allows for flexible selection of marker genes that are tailored to any input dataset.

Our approach relies fundamentally on amino acid sequence comparisons. Translation provides a natural compression algorithm, which has several advantages. First, the amino acid sequences have a third of the length of the corresponding nucleotide sequence. Because the length of the input sequences is a major contributor to the computational complexity of MSA, this reduction in length significantly improves performance and scalability [28, 29]. Additionally, amino acid sequences have a higher degree of conservation relative to nucleotide sequences [30]. This enables us to leverage redundancy in the codon code to more accurately identify orthologous genes and generate more accurate alignments. This enables a more robust and rapid approach for identifying and aligning orthologous genes, especially when applied to phylogenetically diverse datasets.

When performing computationally intensive procedures, amino acid sequences have many advantages; however, nucleotide alignments may be preferable for some applications. To address this, we provide the optional functionality to map the corresponding codons back to the amino acid alignment to return the nucleotide alignment. This can substantially increase the sequence variation contained in the alignment, which may be useful for applications where it is important to distinguish between closely related isolates. Additionally, we leverage the information contained in the amino acid sequences to produce a codon aware alignment. This allows for greater accuracy in placement of functional residues within the gene sequence and reduces the potential for misalignment of codons that may occur when aligning nucleotide sequences.

An important feature of our algorithm is that it relies only on annotated whole genome assemblies, which provides several advantages over commonly used techniques of aligning raw sequencing reads to a reference genome. First, with respect to the size of the files, assemblies are a small fraction of the files containing the raw sequencing data. Second, cognac does not require selection of a reference genome. Different choices of reference genome have been shown to have large influences on the quality of the output alignment, potentially amplifying the frequency of mapping errors [31]. Additionally, the mapping accuracy is severely compromised when considering diverse datasets, even within a species. This limits the application of this method to diverse datasets. Finally, since our approach relies on assemblies, this enables us to analyze genomes sequenced on different platforms, allowing for increased sample size.

Other assembly based methods for estimating the genomic distance between genomes use dimensionality reduction techniques such as k-mers or the MinHash algorithm to estimate the distance between genomes [32, 33]. These methods have the advantage that they can leverage non-coding regions as a source of additional variation; however, the natural structure of the data is lost. Our method not only allows for an estimation of the genetic distances between isolates, but also produces an alignment that can be used in downstream applications. This has the potential to leverage the alignment to identify recombinogenic genes, and has the potential for use in gaining biological insights into molecular evolution.

Our algorithm was able to scale to extremely large datasets. For a data set of 11,639 Escherichia genomes we were able to generate a neighbor joining tree from a nucleotide concatenated gene alignment in less than 17 h. This is accomplished by reducing the computational overhead of MSA in two ways: (1) translating the sequences, effectively reducing their length; and (2) reducing the number of sequences by only aligning unique alleles. For extremely large datasets, this results in an approximately 99% reduction in the number of sequences that need to be aligned, allowing for great improvements in scalability, and allows for application to extremely large datasets.

## Conclusions

In summary, cognac is a robust, rapid method for generating concatenated gene alignments that scales to extremely large datasets. Our method uses a data driven approach for identification of phylogenetic markers, which are efficiently aligned and concatenated into a single alignment for downstream phylogenetic analysis. The pipeline is open source and freely available as an R package. We expect our tool will be generally useful for many different types of analysis and will enable evolutionary insights in a broad range of applications.

## Availability and requirements

- Project Name: cognac
- Project Home page: https://github.com/rdcrawford/cognac
- Operating system: Tested on Linux
- Programming languages: R, C +  + 
- Other requirements: R 3.6 or higher, CD-HIT (version 4.7), and MAFFT (v7.310).
- License: GNU General Public License, version 2
- Any restrictions to use by non-academics: none

## Supplementary information

**Additional file 1:** Genomes used in this study. Associated meta-data of the genome sequences that were used in this manuscript.

## Data Availability

Genomes for this study were downloaded from the Pathosystems Resource Integration Center (PATRIC) [34], and are available from https://www.patricbrc.org/. All available genomes from the genera of interest available as of 06/01/2020 that were isolated from humans and met the criteria for good quality were downloaded from the PATRIC FTP server. Quality was assessed for completeness, contamination, coarse consistency, and fine consistency via the CheckM algorithm within the PATRIC genome annotation service [35, 36]. Additional genomes used in this study were collected as part of a longitudinal study of carbapenem resistant organisms and are available from RefSeq under BioProject PRJNA603790 and PRJNA690239 [37]. All genome annotations were generated with RAST [17]. cognac source code is available at https://github.com/rdcrawford/cognac. Scripts used in benchmarking are available at https://github.com/rdcrawford/cognac_paper. Additionally, a docker image is available at https://hub.docker.com/repository/docker/rdcrawford/cognac.

## Abbreviations

MSA: Multiple sequence alignment
ML: Maximum likelihood

## References

[1] Yang Z, Rannala B (2012). Molecular phylogenetics: principles and practice. Nat Rev Genet.

[2] Kreitman M (1983). Nucleotide polymorphism at the alcohol dehydrogenase locus of *Drosophila melanogaster*. Nature.

[3] Edwards SV (2009). Is a new and general theory of molecular systematics emerging?. Evolution.

[4] Galtier N, Daubin V (2008). Dealing with incongruence in phylogenomic analyses. Philos. Trans. R. Soc. B Biol. Sci..

[5] Rokas A (2005). Animal evolution and the molecular signature of radiations compressed in time. Science.

[6] Ciccarelli FD (2006). Toward automatic reconstruction of a highly resolved tree of life. Science.

[7] Philippe H, Lartillot N, Brinkmann H (2005). Multigene analyses of bilaterian animals corroborate the monophyly of *Ecdysozoa*, *Lophotrochozoa*, and *Protostomia*. Mol Biol Evol.

[8] Zhu Q (2019). Phylogenomics of 10,575 genomes reveals evolutionary proximity between domains Bacteria and Archaea. Nat Commun.

[9] Olmstead RG, Sweere JA (1994). Combining data in phylogenetic systematics: an empirical approach using three molecular data sets in the solanaceae. Syst Biol.

[10] Leigh JW, Susko E, Baumgartner M, Roger AJ (2008). Testing congruence in phylogenomic analysis. Syst Biol.

[11] Tonini J, Moore A, Stern D, Shcheglovitova M, Ortí G. Concatenation and species tree methods exhibit statistically indistinguishable accuracy under a range of simulated conditions. PLoS Curr. **7** (2015). 10.1371/currents.tol.34260cc27551a527b124ec5f6334b6be PMC4391732 25901289

[12] Gadagkar SR, Rosenberg MS, Kumar S (2005). Inferring species phylogenies from multiple genes: Concatenated sequence tree versus consensus gene tree. J. Exp. Zoolog. B Mol. Dev. Evol..

[13] Rajendhran J, Gunasekaran P (2011). Microbial phylogeny and diversity: small subunit ribosomal RNA sequence analysis and beyond. Microbiol Res.

[14] Maiden MCJ (1998). Multilocus sequence typing: a portable approach to the identification of clones within populations of pathogenic microorganisms. Proc Natl Acad Sci U S A.

[15] Segata N, Börnigen D, Morgan XC, Huttenhower C (2013). PhyloPhlAn is a new method for improved phylogenetic and taxonomic placement of microbes. Nat Commun.

[16] Page AJ (2015). Roary: rapid large-scale prokaryote pan genome analysis. Bioinformatics.

[17] Aziz RK (2008). The RAST server: rapid annotations using subsystems technology. BMC Genomics.

[18] Seemann T (2014). Prokka: rapid prokaryotic genome annotation. Bioinforma Oxf Engl.

[19] Hyatt D (2010). Prodigal: prokaryotic gene recognition and translation initiation site identification. BMC Bioinformatics.

[20] Fu L, Niu B, Zhu Z, Wu S, Li W (2012). CD-HIT: accelerated for clustering the next-generation sequencing data. Bioinformatics.

[21] Katoh K, Standley DM (2013). MAFFT multiple sequence alignment software version 7: improvements in performance and usability. Mol Biol Evol.

[22] Eddelbuettel D, Francois R (2011). Rcpp: seamless R and C++ integration. J Stat Softw.

[23] Robison AD, Padua D (2011). Intel® Threading Building Blocks (TBB). Encyclopedia of Parallel Computing.

[24] Bengtsson H, R Core Team. future.apply: Apply Function to Elements in Parallel using Futures. 2020.

[25] Paradis E, Claude J, Strimmer K (2004). APE: analyses of phylogenetics and evolution in R language. Bioinformatics.

[26] Dettman JR, Sztepanacz JL, Kassen R. The properties of spontaneous mutations in the opportunistic pathogen *Pseudomonas aeruginosa*. BMC Genomics 2016;17. 10.1186/s12864-015-2244-3 PMC4702332 26732503

[27] Porter NT (2020). Phase-variable capsular polysaccharides and lipoproteins modify bacteriophage susceptibility in *Bacteroides thetaiotaomicron*. Nat Microbiol.

[28] Katoh K, Rozewicki J, Yamada KD (2019). MAFFT online service: multiple sequence alignment, interactive sequence choice and visualization. Brief Bioinform.

[29] Wang L, Jiang T (1994). On the complexity of multiple sequence alignment. J Comput Biol.

[30] Koonin EV, Galperin MY (2003). Principles and methods of sequence. Analysis sequence - evolution - function: computational approaches in comparative genomics.

[31] Bush SJ (2020). Genomic diversity affects the accuracy of bacterial single-nucleotide polymorphism–calling pipelines. GigaScience.

[32] Lees JA (2019). Fast and flexible bacterial genomic epidemiology with PopPUNK. Genome Res.

[33] Ondov BD (2016). Mash: fast genome and metagenome distance estimation using MinHash. Genome Biol.

[34] Gillespie JJ (2011). PATRIC: the comprehensive bacterial bioinformatics resource with a focus on human pathogenic species. Infect Immun.

[35] Parks DH, Imelfort M, Skennerton CT, Hugenholtz P, Tyson GW (2015). CheckM: assessing the quality of microbial genomes recovered from isolates, single cells, and metagenomes. Genome Res.

[36] Parrello B (2019). A machine learning-based service for estimating quality of genomes using PATRIC. BMC Bioinformatics.

[37] Hayden MK (2015). Prevention of colonization and infection by *Klebsiella pneumoniae* carbapenemase-producing *enterobacteriaceae* in long-term acute-care hospitals. Clin Infect Dis Off Publ Infect Dis Soc Am.

